# Hepatitis E Virus Genotype 4 Outbreak, Italy, 2011

**DOI:** 10.3201/eid1901.120983

**Published:** 2013-01

**Authors:** Anna R. Garbuglia, Paola Scognamiglio, Nicola Petrosillo, Claudio Maria Mastroianni, Pasquale Sordillo, Daniele Gentile, Patrizia La Scala, Enrico Girardi, Maria R. Capobianchi

**Affiliations:** Lazzaro Spallanzani National Institute for Infectious Diseases, Rome, Italy (A.R. Garbuglia, P. Scognamiglio, N. Petrosillo, P. La Scala, E. Girardi, M.R. Capobianchi);; Sapienza University, Rome (C.M. Mastroianni);; Santa Maria Goretti Hospital, Latina, Italy (C.M. Mastroianni);; Tor Vergata University Polyclinic, Rome (P. Sordillo);; and Local Health Unit ASL Roma H, Rome (D. Gentile)

**Keywords:** Hepatitis E virus, outbreak, zoonosis, genotype, Italy, viruses, outbreak, hepatitis, HEV, RNA, PCR

## Abstract

During 2011, 5 persons in the area of Lazio, Italy were infected with a monophyletic strain of hepatitis E virus that showed high sequence homology with isolates from swine in China. Detection of this genotype in Italy parallels findings in other countries in Europe, signaling the possible spread of strains new to Western countries.

Hepatitis E virus (HEV) represents the major etiologic agent of enterically transmitted, non-A, non-B hepatitis. One third of the world population is estimated to have been infected with HEV, although the global extent of infection is unknown (http://apps.who.int/gb/ebwha/pdf_files/A62/A62_22-en.pdf). Recent evidence indicates that the family *Hepeviridae* may contain several genera and that viruses from some genera can be transmitted from animals to humans and vice versa (*1*). Gene sequence analysis demonstrates that HEV isolates are divided into 4 genotypes and >24 subgenotypes (*2*). Increasing evidence shows that genotypes 3 and 4 are zoonotic, with domestic pigs a likely reservoir of infection; these genotypes have also been found in boars and deer (*3*).

HEV genotype 4 is endemic among humans in China, Japan, India, and Indonesia (*4*) and was detected during 2008 from swine fecal samples in Belgium (*5*). Human infections with imported strains of this genotype that later became endemic have subsequently been described in Germany (*6*) and northern France (*7*). Recently, multiple cases of HEV infection have been described in southern France (*8*,*9*).

In different areas of Italy, HEV seroprevalence estimates range from 1% to 6% (*10*); prevalence is 2.9% in the Lazio region and 2.5% in the province of Rome (*11*). However, the number of acute hepatitis cases caused by HEV reported in Italy is relatively low compared with surrounding European countries and is probably underestimated; most cases are travel-related and caused by genotype 1, but sporadic cases spread within Italy have been caused by genotype 3 (*12*). We report an outbreak of HEV genotype 4 infection among persons living in Lazio, an administrative region of Italy that encompasses Rome.

## The Study

During March and April 2011, diagnoses of acute HEV infection were made for diagnosed for 5 patients admitted to 3 hospitals in Lazio: 2 in Rome and 1 in Latina. Diagnosis was made on the basis of clinical and laboratory signs of acute hepatitis and detection of IgG and IgM against HEV by immunoassay (Radim S.P.A., Rome, Italy); Other causes of acute liver injury, including drug toxicity, autoimmune hepatitis, and infection by hepatitis A, B, and C viruses, as well as by cytomegalovirus and Epstein-Barr virus, were excluded (Table 1). All patients recovered rapidly with supportive care.

**Table 1:** Clinical and demographic characteristics of 5 case-patients involved in outbreak of HEV infection, Lazio, Italy, March–April 2011*

Characteristic	Case-patient 1	Case-patient 2	Case-patient 3	Case-patient 4	Case-patient 5
HEV isolate no.	E2104	E2105	E2107	E2111	E2115
Age, y/sex	60/M	38/M	66/M	57/M	74/M
Date of symptom onset	Mar 10	Mar 12	Mar 14	Apr 1	Apr 2
Date of first HEV Ab detection	Mar 19	Mar 23	Mar 25	Apr 11	Apr 14
Clinical signs, symptoms	Fatigue, jaundice, dark urine	General malaise, fatigue	Fatigue, dark urine	Jaundice	Fatigue, vomiting
Duration of hospitalization, d	18	25	14	13	16
Alanine aminotransferase, IU/L†	4,017	2,648	2,648	5,163	3,001
Total bilirubin, mg/dL‡	6.5	20.0	15.6	14.7	8.0
Hepatitis A virus IgG	Positive	Negative	Negative	Positive	Positive
HBV surface antigen	Negative	Negative	Negative	Negative	Negative
HBV core Ab	Negative	Negative	Negative	Positive	Negative
Hepatitis C virus Ab	Negative	Negative	Negative	Negative	Negative
HIV Ab	Negative	Negative	Negative	Negative	Negative
Cytomegalovius IgM	Negative	Negative	Negative	Negative	Negative
EBV EA IgG	Negative	Negative	Negative	Negative	Negative
EBV VCA IgM	Negative	Negative	Negative	Negative	Negative

*HEV, hepatitis E virus; Ab, antibody; HBV, hepatitis B virus; EBV, Epstein–Barr virus; EA, early antigen; VCA, viral capsid antigen. †Reference range 7–34 IU/L. ‡Reference range 0.3–1.2 mg/dL.

**Figure 1:**
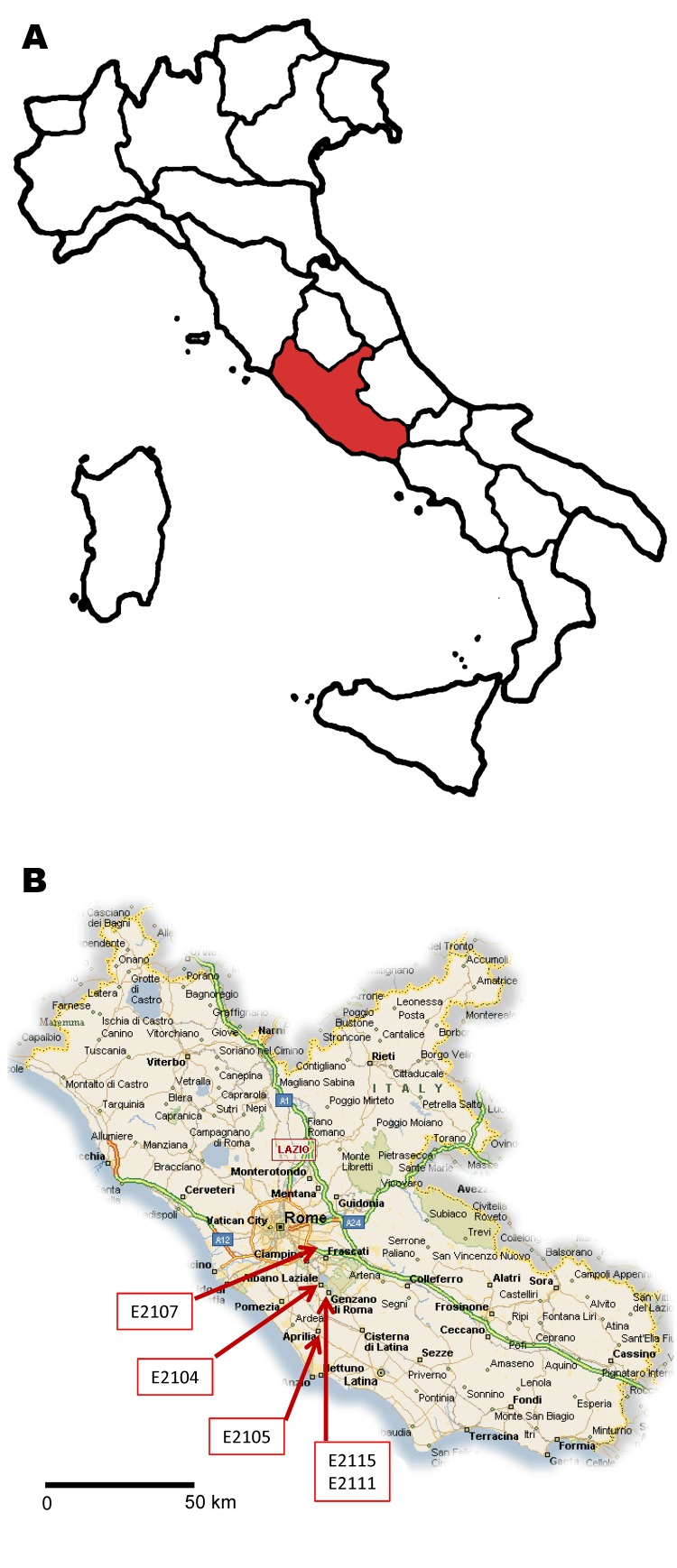
A) Lazio region of Italy (red). B) Residence location of 5 cases of hepatitis E virus (HEV) subtype 4d infection involved in an autochthonous outbreak, March–April 2011. (Map source: CartineGeografiche, Catania, Sicily, Italy; www.cartinegeografiche.eu).

The 5 case-patients lived in the same area (maximum distance apart: 27 km) (Figure 1). The cases were reported to the health authorities, who conducted a structured interview to determine travel history and other risk factors (Table 2). None of the case-patients reported recent travel to disease-endemic areas, and relationships among them or exposure to a common source were not identified. It was not possible to conduct the complete interview for 1 patient (isolate no. E2107).

**Table 2:** Exposure and potential risk factors for 4 patients infected with hepatitis E virus during outbreak in Lazio, Italy, March–April 2011*

Factor	Case-patient 1	Case-patient 2	Case-patient 4	Case-patient 5
Underlying disease	IHD	None	IHD	IHD
Farm residence or employment	No	No	No	No
Manure used for fruits/vegetables grown in backyard	No	No	No	No
Professional or amateur hunter	No	No	Yes	No
Occupational exposure to animals	No	No	No	No
Household pet or domestic animal ownership	Dogs	No	Dogs, chicken	No
Risk behavior during previous 2 months				
Received blood transfusion	No	No	No	No
Traveled abroad	No	London	No	No
Had close contact with a recent traveler	Yes†	No	No	No
Visited a farm or petting zoo	No	No	No	No
Drank water from a well	No	No	No	No
Had contact with surface water	No	No	No	No
Had contact with waste water	Yes	No	No	No
Food products consumed during previous 2 months				
Mussels	Yes	No	Yes	No
Shellfish	Yes	Yes	Yes	No
Poultry, undercooked	No	No	No	No
Pork products, raw or undercooked	No	Yes	Yes	Yes
Pork meat, undercooked	No	No	No	No
Horse meat	No	No	No	No
Beef, raw or undercooked	No	No	Yes	Yes
Wild boar, cooked or undercooked	No	No	Yes	No
Wild-animal meat (other than wild boar)	No	No	No	No
Cattle liver	No	No	No	Yes
Pig liver	No	No	No	Yes
Other (e.g., kidney, gut)	No	No	No	No

*Complete information was not available for case-patient 3 (isolate no. E2107). IHD, ischemic heart disease. †Contact with a person returning from Poland and Romania.

**Figure 2:**
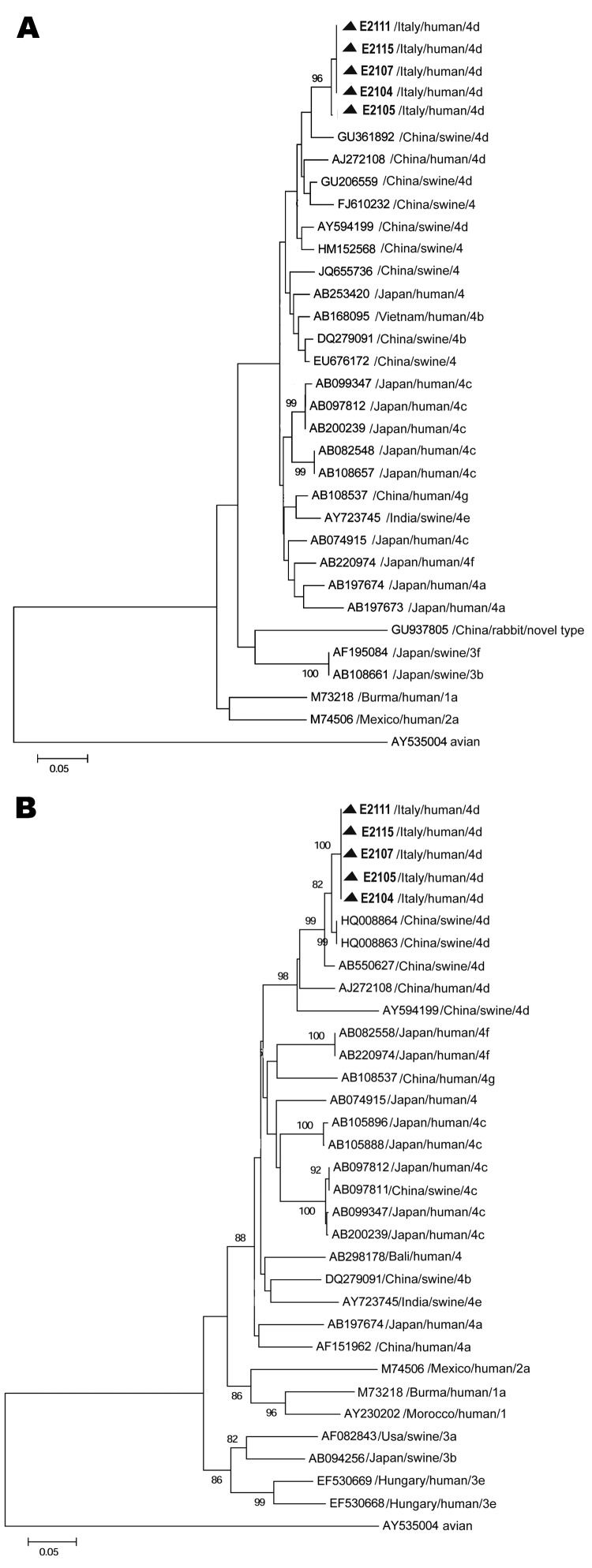
Phylogenetic trees based on partial open reading frame (ORF) sequences of the hepatitis E virus monophyletic strain involved in an outbreak in Lazio, Italy, March–April 2011. A) ORF1, 172 nt. Sequences from the outbreak in Italy could not be submitted to GenBank, being <200 nt long; they are available on request from the authors. B) ORF2, 411 nt. The ORF 2 sequence (identical in all 5 patients) described in this panel was submitted to GenBank (accession no. JX401928). Neighbor-joining trees were built by using MEGA5.1 software (www.megasoftware.net), applying the Jukes-Cantor p-distance model of nucleotide substitution. Bootstrap values were determined on 1,000 resamplings of the data set; bootstrap values >80 are shown. Reference strains from GenBank are also included in the trees. Reference viral strains are identified by GenBank accession number, source, country of origin, and respective genotype and subtype. The avian strain AY535004 was used as outgroup. Triangles indicate sequences recovered during the outbreak in Italy. Scale bars represent nucleotide substitutions per site.

Serum samples were tested for RNA by reverse transcription-nested PCR by using primers designed within open reading frame (ORF) 1 and ORF2 (*12*,*13*). The sequence data from these genetic regions identified a monophyletic strain belonging to genotype 4, subgenotype d. ORF1 nucleotide sequences (172 bp) from the outbreak showed high similarity among patients (99.2%) and 96% and 95% identity with HEV4d swine hb-3 and human T1 isolates from China, respectively (GenBank accession nos. GU361882 and AJ272108). Sequences identified in the ORF2 region (411 bp) in samples from the 5 case-patients showed 100% similarity. The sequences also were closely related to the strains KMsw-3 (nucleotide similarity 98.5%; accession no. HQ008864) and KMsw-1 (98.3%; accession no. HQ008863) isolated from swine and to the strain GS-NJ-13 isolated from humans (97.8%; accession no. JF309220.1), all of which originated in China. Figure 2 shows phylogenetic trees for ORF1 and ORF2 partial gene sequences.

The similarity of the strain from this outbreak to strains from recent autochthonous HEV genotype 4 infections in Europe was relatively low: 73.5% in the overlapping 37–172-nt ORF1 region of the isolate from Germany (GenBank accession no. EU879120) (*6*), 85.7% in the overlapping 31–373-nt ORF2 region of the isolate from northern France (accession no. GU982294) (*7*), and 84.7%–84.8% in the 329-nt overlapping ORF2 region of 5 isolates from southern France (*9*). Consistent with these data, 85.1% and 85.3% identity was observed in the 537-nt ORF2 region of 2 of the isolates from southern France (accession nos. JF900631 and JF900632) (*8*), (Technical Appendix).

## Conclusions

An outbreak of HEV infection caused by genotype 4 in Italy involved 5 case-patients who lived in the same area and did not travel to disease-endemic areas. The high genetic similarity in ORF1 and ORF2 among the 5 HEV isolates supports a point-source outbreak and not sustained local circulation of this strain. Epidemiologic information did not identify the transmission route; available data ruled out direct transmission among patients, and parenteral transmission is unlikely because none of the patients had received blood transfusions, tattoos, or drug injections.

Three patients had IgG against hepatitis A virus (HAV); no information on HAV vaccination status was available. However, the presence of these antibodies does not necessarily imply higher than average levels of exposure to enterically transmitted viruses; a HAV seroprevalence >60% has been reported in persons in the birth cohorts of the 5 case-patients in central Italy (*14*).

Consumption of contaminated food (i.e., pork or wild animal meat, bivalve mollusks, or shellfish) is considered the most likely source of infection with HEV genotype 3 in Europe (*8*,*12*). For genotype 4, uncooked deer meat was indicated as a source of human infection in Japan (*15*), and undercooked pork meat was the probable source of infection in southern France (*8*,*9*). The isolates from this study had the highest genetic similarity to subgenotype 4d strains of swine origin from China (Figure 2), which suggests a possible zoonotic origin through either direct (e.g., ingestion of raw or undercooked pork products) or indirect (e.g., by water contaminated with animal excreta) transmission.

Possible sources of infection with this HEV strain that cannot be ruled out include contaminated food from abroad and direct introduction through infected immigrants from China or other countries in Asia. However, the proportion of immigrants from Asia in this area of Italy, 0.77%, is lower than the national average (1.26%; www.comuni-italiani.it/statistiche/stranieri.html). Available data do not support correlations between immigration from China and spread of this HEV genotype in Lazio.

Strong sequence similarity (>96%) was observed between HEV isolates from human cases in northern and southern France and the strain isolated from swine in Belgium (*5*), classified as subgenotype 4b. Human infection with HEV genotype 4 reported in Germany in 2008 (*6*) was attributed to a different subgenotype (4f). The strain involved in the outbreak in Italy showed relatively poor genetic resemblance with any of these strains, which indicates that different HEV genotype 4 strains have been recently introduced in Europe.

In summary, this outbreak of HEV genotype 4 infection in Italy was not linked to infection by imported foods or persons traveling from endemic areas, which suggests the possibility that newly imported strains might spread this virus to new areas. Molecular characterization of HEV outbreaks in Europe is needed to implement epidemiologic mapping of infection with introduced strains of HEV and subsequent circulation.

Technical Appendix Methods used to generate partial open reading frame 2 sequences, including the 537-nt region of sequences from southern France described by Colson et al. (*1*).

## References

[1] Drexler JF, Seelen A, Corman VM, Fumie Tateno A, Cottontail V, Melim Zerbinati R, Bats worldwide carry hepatitis E virus-related viruses that form a putative novel genus within the family *Hepeviridae.* J Virol. 2012;86:9134–47. 10.1128/JVI.00800-12 22696648 PMC3416139

[2] Lu L, Li C, Hagedorn CH. Phylogenetic analysis of global hepatitis E virus sequences: genetic diversity, subtypes and zoonosis. Rev Med Virol. 2006;16:5–36. 10.1002/rmv.482 16175650

[3] Kamar N, Bendall R, Legrand-Abravanel F, Xia NS, Ijaz S, Izopet J, Hepatitis E. Lancet. 2012;379:2477–88. 10.1016/S0140-6736(11)61849-7 22549046

[4] Hu GD, Ma X. Detection and sequences analysis of bovine hepatitis E virus RNA in Xinjiang autonomous region. Bing Du Xue Bao. 2010;26:27–32. 20329555

[5] Hakze-van der Honing RW, van Coillie E, Antonis AF, van der Poel WH. First isolation of hepatitis E virus genotype 4 in Europe through swine surveillance in the Netherlands and Belgium. PLoS ONE. 2011;6:e22673. 10.1371/journal.pone.0022673 21829641 PMC3148228

[6] Wichmann O, Schimanski S, Koch J, Kohler M, Rothe C, Plentz A, Phylogenetic and case-control study on hepatitis E virus infection in Germany. J Infect Dis. 2008;198:1732–41. 10.1086/593211 18983248

[7] Tessé S, Lioure B, Fornecker L, Wendling MJ, Stoll-Keller F, Bigaillon C, Circulation of genotype 4 hepatitis E virus in Europe: First autochthonous hepatitis E infection in France. J Clin Virol. 2012;54:197–200. 10.1016/j.jcv.2012.02.007 22405947

[8] Colson P, Romanet P, Moal V, Borentain P, Purgus R, Benezech A, Autochthonous infections with hepatitis E virus genotype 4, France. Emerg Infect Dis. 2012;18:1361–4. 10.3201/eid1808.111827 22840196 PMC3414032

[9] Colson P, Swiader L, Motte A, Ferretti A, Borentain P, Gerolami R. Circulation of almost genetically identical hepatitis E virus of genotype 4 in France. J Clin Virol. 2012;55:181–3. Epub 2012 Jul 25. 10.1016/j.jcv.2012.06.014 22835777

[10] Romanò L, Paladini S, Tagliacarne C, Canuti M, Bianchi S, Zanetti AR. Hepatitis E in Italy: a long-term prospective study. J Hepatol. 2011;54:34–40. 10.1016/j.jhep.2010.06.017 20888660

[11] Vulcano A, Angelucci M, Candelori E, Martini V, Patti AM, Mancini C, HEV prevalence in the general population and among workers at zoonotic risk in Latium Region. Ann Ig. 2007;19:181–6. 17658105

[12] La Rosa G, Muscillo M, Vennarucci VS, Garbuglia AR, La Scala P, Capobianchi MR. Hepatitis E virus in Italy: molecular analysis of travel-related and autochthonous cases. J Gen Virol. 2011;92:1617–26. 10.1099/vir.0.031278-0 21471314

[13] Mizuo H, Suzuki K, Takikawa Y, Sugai Y, Tokita H, Akahane Y, Polyphyletic strains of hepatitis E virus are responsible for sporadic cases of acute hepatitis in Japan. J Clin Microbiol. 2002;40:3209–18. 10.1128/JCM.40.9.3209-3218.2002 12202555 PMC130758

[14] Ansaldi F, Bruzzone B, Rota MC, Bella A. Ciofi degli Atti M, Durando P, Gasparini R, Icardi G; Serologic Study Group. Hepatitis A incidence and hospital-based seroprevalence in Italy: a nation-wide study. Eur J Epidemiol. 2008;23:45–53. 10.1007/s10654-007-9198-y 17978852

[15] Nishizawa T, Takahashi M, Mizuo H, Miyajima H, Gotanda Y, Okamoto H. Characterization of Japanese swine and human hepatitis E virus isolates of genotype IV with 99% identity over the entire genome. J Gen Virol. 2003;84:1245–51. 10.1099/vir.0.19052-0 12692290

