# Supplementary material for: Hepatitis E Virus Genotype 4 Outbreak, Italy, 2011
Source: Emerg Infect Dis. 2013 Jan;19(1):110–4. doi: 10.3201/eid1901.120983 (PMC3558000; doi:10.3201/eid1901.120983)
Supplement: Technical Appendix — Methods used to generate partial open reading frame 2 sequences, including the 537-nt region of sequences from southern France described by Colson et al. (1). [file 12-0983-Techapp-s1.pdf]

Article DOI: http://dx.doi.org/10.3201/eid1901.120983

Hepatitis E Virus Genotype 4 Outbreak, Italy, 2011

Technical Appendix

Methods used to generate partial open reading frame 2 sequences, including the 537-nt region of sequences from southern France described by Colson et al. (*1*).

Amplification

Genotype 4-specific set of PCR primers listed in Technical Appendix Table were used. The first step of PCR was performed with FastStart Taq DNA Polymerase (Roche Molecular Diagnostics, Somerville, CA, USA), using 32SEN1 and 0RF2ANTISENSE primers. The PCR conditions comprised an initial denaturation at 94°C for 15 min; followed by 35 cycles at 94°C for 45 s, 50°C for 30s, and 72°C for 45s; and a final extension at 72°C for 7 min. Five microliters of the first PCR products were used as template in the second-round PCR using 32SEN2 and 0RF2ANTISENSE primers. The second-round PCR conditions were an initial denaturation at 98°C; followed by 35 amplification cycles each comprising denaturation (94°C for 30s), annealing (58°C for 30s), and extension (72°C for 45s); and a final extension at 72°C for 7 min.

Second-round PCR products were sequenced with the ABI Prism 310 Genetic Analyzer (Applied Biosystems, Foster City, CA, USA), using the BigDye Terminator Cycle Sequencing Kit (Applied Biosystems). The sequences were aligned by using CLUSTALW X1.5 software (http://npsa-pbil.ibcp.fr/)

Reference

1. Colson P, Romanet P, Moal V, Borentain P, Purgus R, Benezech A, et al. Autochthonous infections with hepatitis E virus genotype 4, France. Emerg Infect Dis. 2012;18:1361–4. PubMed http://dx.doi.org/10.3201/eid1808.111827

Technical Appendix Table. PCR primers

Primer name	Primer sequence, 5′→ 3′	Nucleotide position on AJ272108 sequence (HEV genotype 4)
32SEN1	CTC AGC CAA TGG CGA GCT GAC AGT	6387–6410
32SEN2	ACA CTT CAG TCG AGA ACG CTC	6419–6439
0RF2ANTISENSE	CGC GAG CAG GGT AGT CAG CGG TA	7017–7039

*HEV, hepatitis E virus.
